# Does acupuncture have advantages in the rehabilitation of vascular mild cognitive impairment? A systematic review and meta-analysis

**DOI:** 10.1016/j.heliyon.2023.e18955

**Published:** 2023-08-09

**Authors:** Zhitao Hou, Xiaodi Yu, Jing Chen, Jacob S. Brenner, Zhongren Sun, Hongcai Shang

**Affiliations:** a College of Basic Medical and Sciences, Heilongjiang University of Chinese Medicine, Harbin, Heilongjiang, 150040, China; b Key Laboratory of Chinese Internal Medicine of the Ministry of Education, Dongzhimen Hospital Affiliated with Beijing University of Chinese Medicine, Beijing, 100700, China; c Department of Systems Pharmacology and Translational Therapeutics, Perelman School of Medicine, University of Pennsylvania, Philadelphia, PA, 19104, USA; d The First Affiliated Hospital of Harbin Medical University, Harbin, Heilongjiang, 150001, China; e Heilongjiang Academy of Traditional Chinese Medicine, Harbin, Heilongjiang, 150036, China; f School of Acupuncture-Moxibustion and Tuina, Heilongjiang University of Chinese Medicine, Harbin, Heilongjiang, 150010, China

**Keywords:** Acupuncture, Electroacupuncture, Vascular, Stroke, Cognitive impairment, Systematic review, Meta-analysis

## Abstract

**Background:**

Vascular mild cognitive impairment (VMCI) is a common impairment caused by vascular factors. VMCI often occurs after stroke, and it is the main clinical manifestation of long-term disability. Many patients are treated with acupuncture in combination with other therapies. However, evidence regarding the effectiveness of this treatment regimen is lacking.

**Aims:**

This meta-analysis aimed to evaluate the efficacy of acupuncture therapy for treating VMCI.

**Methods:**

This systematic review was conducted in accordance with the preferred reporting and meta-analysis guidelines. The CNKI, Wanfang, VIP, CBM, Cochrane Library, PubMed and Embase databases were searched from inception to August 20, 2022. After two researchers independently screened the literature, they extracted the data and evaluated the risk of bias in the included studies. Revman 5.3 software was used for the meta-analysis.

**Summary of review:**

Thirty-two randomized controlled trials (RCTs) were included. The overall effective rate of acupuncture for treating VMCI was 3.06, 95% CI [2.39, 3.91], (P < 0.05). Montreal Cognitive Assessment (MoCA), Mini-Mental State Examination (MMSE), Barthel Index and Activities of Daily Living (ADLs) scores significantly differed between the treatment and control groups, with weighted mean differences (WMDs) [95% CI] (P value) of 1.97 [1.44, 2.49] (P < 0.05), 2.02 [1.50, 2.54] (P < 0.05), 5.54 [3.81, 7.28] (P < 0.05), and 3.43 [2.53, 4.33] (P < 0.05), respectively. The overall effective rate of electroacupuncture (EA) for treating VMCI was better than that of the control group (RR = 2.25, 95% CI, [1.13, 4.50], P < 0.05). MoCA, MMSE, Barthel index and ADL scores differed significantly between the treatment and control groups, with WMDs [95% CI] (P value) of 1.79 [1.20, 2.38] (P < 0.05), 1.45 [0.87, 2.03] (P < 0.05), 5.78 [2.38, 9.18] (P < 0.05), and 3.15 [2.15, 4.15] (P < 0.05), respectively. Acupuncture alone and combined with drug therapy were thus superior to drug therapy alone for improving cognitive function. EA also has potential advantages.

**Conclusions:**

Acupuncture combined with another therapy is better than other therapies alone, such as simple drug therapy, for treating VMCI. However, variations in study duration (4–12 weeks) limit us from drawing any definitive conclusions about long-term effects. Therefore, more RCTs with rigorous designs and reasonable treatment and follow-up durations are needed.

Vascular mild cognitive impairment (VMCI) is a type of cognitive impairment caused by vascular diseases, especially the complications of stroke. Studies have shown that 24%–39% of stroke patients develop cognitive dysfunction within 3 months [1]. VMCI seriously affects patients' social functioning and their ability to carry out activities of daily living (ADLs), and it also increases mortality. Therefore, treatment of VMCI is necessary to reduce the burden on patients, families and society. In recent years, the development of new drugs for cognitive impairment has continued to fail, such as the controversy regarding aducanumab [2] and the failure of gantenerumab [3]. Nimodipine, donepezil, and racetam are the main drugs for vascular mild cognitive impairment, but these drugs have unsatisfactory efficacy and serious side effects [4]. They are gradually being eliminated from clinical practice. In addition, regarding nonpharmacological therapies, Malec et al. [5] suggested that cognitive function training (CFT) had a certain effect on the recovery of cognitive function, especially improving patients' orientation, prolonging attention time, mastering specific skills and techniques, strengthening their understanding of things, analysing and dealing with problems, etc. However, traditional CFT is characterized by the lack of systematic arrangements and professional trainers, which leads to considerable limitations in the treatment of VMCI patients.

According to a 2019 World Health Organization report, acupuncture is the most widely used nonpharmacological therapy in traditional and complementary medicine and is widely used in 113 out of 120 countries [6]. Traditional Chinese acupuncture has been used as the main clinical nondrug treatment method for thousands of years, with clear therapeutic advantages [7]. A recent study showed a positive effect of acupuncture in patients with cognitive impairment after stroke [8]. In addition, an experimental study in rats showed that the use of acupuncture mitigated cognitive impairment by inducing oxidative stress and raising Ca^2+^, which was associated with inhibition of nuclear factor NF-κB and its downstream target gene p53 [9]. Acupuncture is widely recognized for its effectiveness and low cost compared to other treatments. In the expert consensus on VMCI management released by the Chinese Stroke Association (CSA) in 2017, the Montreal Cognitive Assessment (MoCA) and Mini-Mental State Examination (MMSE) were selected as outcome indicators to evaluate the effectiveness of acupuncture in promoting cognitive function. Therefore, this meta-analysis adopted the principles and methods of evidence-based medicine to conduct a comprehensive search and evaluation of the relevant literature on acupuncture and moxibustion for VMCI to clarify their effectiveness and provide an evidentiary basis for their clinical application.

## 1 Data and methods

### 1.1 Retrieval strategy

#### 1.1.1 Databases searched

Seven databases were searched in this study: the China National Knowledge Infrastructure (CNKI), Wanfang Data Knowledge Service Platform (Wanfang), VIP Journal Integration Platform (VIP), China Biology Medicine Disc (CBM), Cochrane Library, PubMed and Embase. The date range was from database inception to September 20, 2022.

Keywords The search terms in Chinese included terms related to vascular disease, such as “cerebral ischaemia”, “cerebral infarction”, “cerebral thrombosis”, “cerebral embolism”, “cerebrovascular disease”, and “stroke”; terms related to mild cognitive impairment, such as “mild cognitive impairment” and “cognitive impairment”; and terms related to acupuncture, such as “acupuncture”, “acupuncture point”, “electroacupuncture”, and “needle”. The search terms in English included “acupuncture”, “electroacupuncture”, “vascular”, “stroke”, “cerebrovascular accident”, “CVA”, “cerebral infarction”, “apoplexy”, “mild impairment”, “cognitive impairment”, and “MCI”, among others. However, it should be noted that in this study, the term acupuncture mainly refers to traditional Chinese acupuncture and electroacupuncture therapy based on traditional acupuncture.

### 1.2 Inclusion criteria

#### 1.2.1 Document type

This review was restricted to randomized controlled trials whose protocols explicitly stated that they were randomized or described a means of randomization, such as a random number table, dice rolling, coin flipping, or a lottery. Blinding was not a requirement for inclusion. Only reports in Chinese or English were considered.

#### 1.2.2 Research subjects

The included patients were required to meet the diagnostic criteria for stroke, such as the Chinese Guidelines for the Diagnosis and Treatment of Acute Ischaemic Stroke and the Chinese Guidelines for the Diagnosis and Treatment of Intracerebral Haemorrhage, and to have a diagnosis of stroke (ischaemic stroke or haemorrhagic stroke) on the basis of diagnostic imaging such as CT or MRI. Additionally, the patients were required to meet the criteria for mild cognitive impairment according to the Diagnostic and Statistical Manual of Psychiatry of the American Psychiatric Association, the Expert Consensus on the Management of Cognitive Disorders after Stroke, etc., and to show mild cognitive impairment in domains such as attention, memory, reasoning and language according to tests such as the MMSE and MoCA. The intervention group received acupuncture therapy (including electroacupuncture), acupuncture combined with Western medicine, cognitive training or other basic treatments, while the control group received only nonacupuncture therapy. No restrictions were placed on the age, sex, race or disease course of the subjects.

#### 1.2.3 Outcome indicators

To be included, studies were required to report the clinical response rate, MMSE score, and/or MoCA score.

### 1.3 Exclusion criteria

Studies that met any of the following conditions were excluded: (1) animal experiments; (2) self-controlled studies, case reports, reviews, systematic reviews/meta-analyses, or empirical reports; (3) duplicates of the same article or multiple studies of the same cohort (in the latter case, only the study with the latest and most complete content was included in the analysis); (4) conference papers, dissertations, and literature that could not be obtained in full-text form (journal papers with unavailable content, journal papers with invalid citations, and articles that were actually dissertations or database entries); and (5) studies that used any design other than a randomized controlled trial, applied an incorrect randomization method, or allocated patients on a semirandomized basis such as the admission number or visit number; (6) Literature on nonstroke-associated cognitive dysfunction; (7) Studies that used acupuncture in both the intervention group and the control group; (8) Literature in languages other than Chinese and English.

### 1.4 Literature quality evaluation

The quality assessment tool recommended by the Cochrane Handbook was used to rate six aspects of each study: selection bias, implementation bias, measurement bias, attrition bias, reporting bias and other biases. This study took the reporting of adverse events as the main reference for the evaluation of other biases. Each item is divided into three risk levels: low risk, high risk and uncertain risk. Each risk-of-bias evaluation was independently performed by two persons. If there was any disagreement, a third party made the final decision.

### 1.5 Literature screening and data extraction

The literature was screened strictly according to the inclusion and exclusion criteria. First, the titles and abstracts of the studies were read, and the articles that met the inclusion and exclusion criteria were extracted. Those studies were then screened in full-text form. The literature search and screening were carried out by two researchers independently, and their results were compared. When there was disagreement over a study, the two researchers resolved their disagreement by discussion or referred the decision to a third researcher. After literature screening and selection, the basic features of the included studies were extracted, including study ID (an identifier consisting of the name of the first author and the year of publication), age, sample size, intervention measures, course of treatment, and efficacy indicators, among others. Data entry was carried out by two researchers independently, and the extracted information was stored in a Microsoft Excel database.

### 1.6 Statistical methods

Review Manager 5.2 software was used for the meta-analysis in this study. The main statistical methods involved included the selection of effect size and effect model, heterogeneity analysis, and publication bias analysis.

#### 1.6.1 Effect size and selection of model type

The effect size and model type were selected according to the original study design type and data type. The outcome index data of this study included binary data and continuous data. The weighted mean difference (WMD) was selected for continuous data. For binary data, the relative risk (RR) was used. The 95% confidence intervals (CIs) were also calculated. Models of effects can be divided into fixed-effects and random-effects models. When the heterogeneity among the original studies was small, the fixed-effects model was used. When the heterogeneity among the original studies was large, the random-effects model was used.

#### 1.6.2 Heterogeneity test

In this study, the Q test and I^2^ test were used to analyse the heterogeneity of the studies; the I^2^ test was the more efficient of the two. The results of the Q test are presented as P values, with P < 0.1 indicating heterogeneity among studies. The results of the I^2^ test are presented as percentages. The Cochrane Handbook defines 4 levels of heterogeneity according to the magnitude of I^2^ values: mild heterogeneity (0%–40%), moderate heterogeneity (30%–60%), substantial heterogeneity (50%–90%), and considerable heterogeneity (75%–100%). When P > 0.1 in the Q test and I^2^ ≤ 50% in the I^2^ test, these results indicate that the heterogeneity between studies is small, and the fixed-effects model is selected. When P < 0.1 and I^2^ > 50%, the heterogeneity was considered large; in this event, the random-effects model was selected, and the source of heterogeneity was determined by subgroup analysis and sensitivity analysis. When the P value and I^2^ value had conflicting implications, the I^2^ value was prioritized.

#### 1.6.3 Publication bias

In this study, qualitative and quantitative methods were used to evaluate publication bias. For the qualitative analysis, the funnel plot method was used. The X-axis of the funnel plot represents the effect size of each study, and the Y-axis represents the standard error of the effect size. The combined effect size is taken as the central axis and intersects the X-axis as the vertical line. The points distributed to the left of the vertical line represent studies whose effect sizes are smaller than the combined effect size, and the points distributed to the right of the vertical line represent studies whose effect sizes are larger than the combined effect size. If the numbers of points to the left and right of the vertical line are roughly equal, publication bias is considered to be absent; if the numbers of points to the left and right of the vertical line are significantly different, publication bias is considered to be present. Egger's and Begg's tests were used for quantitative analysis. When P > 0.05, the possibility of bias was considered small.

## 2 Results

### 2.1 Literature search and screening

**Fig. 1:**
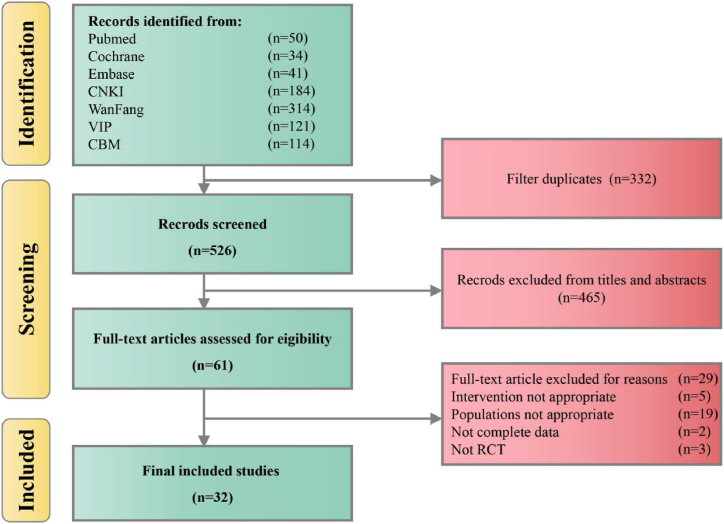
Flowchart of literature screening.

A total of 858 articles were retrieved through computerized database searches. The studies retrieved from Chinese-language databases comprised 184 articles from CNKI, 314 articles from Wanfang, 121 articles from VIP and 114 articles from CBM. The studies retrieved from English-language databases comprised 50 articles from PubMed, 34 articles from the Cochrane Library, and 41 articles from Embase. EndNote 19 literature citation management software was used to combine all the citations into a single database automatically; this list was then manually screened to remove duplicates. Furthermore, the titles and abstracts were read to screen out any studies that were not related to the aim of this review. Finally, the full-text versions of the remaining articles were read and screened based on the inclusion and exclusion criteria. Ultimately, 32 studies were included in this review. A flow chart of the literature screening process is shown in Fig. 1.

### 2.2 Basic characteristics of the included studies

All 32 of the included studies reported basic information on their subjects. Regarding sample size, 1410 cases were included in the experimental group, and 1398 cases were included in the control group, with the sample size ranging from 14 to 94 cases per study in both groups. Electroacupuncture was used in 10 studies, and ordinary acupuncture was used in 22 studies. The control groups of 21 studies received Western drugs, including atorvastatin in 2 studies, donepezil in 3 studies, ganglioside in 1 study, and nimodipine in 15 studies. The course of treatment lasted for 2–12 weeks. In terms of efficacy indicators, 17 studies used the total effective rate, 21 studies used MoCA scores, 22 studies used MMSE scores, 8 studies used quality of life as evaluated by the ADL scale, and another 6 studies used the Barthel index. The basic characteristics are presented in detail in Table 1.

**Table 1:** Basic characteristics of the included studies.

Study ID	Sample size		Interventions		Acupoints	Subject age (years)		Period of treatment	Outcomes
	Test	Control	Test	Control		Test	Control		
Yu Bao 2012 [10]	30	30	Acupuncture	Donepezil	Baihui (DU20), Sishencong (EX-HN1), Fengchi (GB20), Shenmen (HT7), Sanyinjiao (SP6), Taixi (KI3), Xuanzhong (GB39)	63 ± 6	64 ± 6	8 w	①,③, ⑤
Linlin Deng 2016 [11]	40	39	Acupuncture	Nimodipine	Baihui (DU20), Sishencong (EX-HN1), Shuigou (DU26), Sibai (ST2), Fengchi (GB20), Wangu (GB12), Tianzhu (BL10), Shenmen (HT7), Neiguan (PC6), Taichong (LR3), Sanyinjiao (SP6), Fenglong (ST40)	63.85 ± 7.53	60.69 ± 6.47	12 w	①, ②
Hong Zhang 2013 [12]	80	75	Electroacupuncture	Nimodipine	Baihui (DU20), Sishencong (EX-HN1), Shenting (DU24)	71 ± 8	72 ± 9	8 w	①, ③
Huang Li 2021 [13]	60	60	Electroacupuncture	Sham acupuncture	Baihui (GV20), Yintang (GV29), Shenting (GV24), Shuigou (GV26), Naohu (GV17), Sishencong (EX-HN1), Fengchi (GB20), Shenmen (HT7), Sanyinjiao (SP6)	65.1 ± 7.5	64.6 ± 8.4	8 w	②, ③
Jiang Cai 2016 [14]	52	51	Acupuncture + Cognitive training	Cognitive training	Baihui (DU20), Shenting (DU24)	62.33 ± 7.72	62.37 ± 7.89	12 w	②, ③
Li Guohui 2009 [15]	58	57	Acupuncture	Nimodipine	Sishencong (EX-HN1), Benshen (BG13), Baihui (DU20), Fengchi (GB20)	54.14 ± 7.43	54.49 ± 8.06	12 w	③
Li Lanchen 2019 [16]	34	34	Acupuncture + Nimodipine	Nimodipine	Baihui (DU20), Sishencong (EX-HN1), Shenting (DU24), Benshen (BG13), Fengchi (GB20), Neiguan (PC6), Shenmen (HT7), Touwei (ST8), Hegu (LI4), Quchi (LI11), Zusanli (ST36), Wangu (GB12), Sanyinjiao (SP6), Taichong (LR3), Taixi (KI3)			12 w	①, ⑤
Li Wei 2012 [17]	48	46	Electroacupuncture + Nimodipine	Nimodipine	Baihui (DU20), Shenting (DU24), Quchi (LI11), Sishencong (EX-HN1), Fengchi (GB20)	68.29 ± 8.22	69.22 ± 7.88	12 w	①,③, ⑤
Liu Jialin 2015 [18]	60	60	Acupuncture + Nimodipine	Nimodipine	Baihui (DU20), Sishencong (EX-HN1), Shuigou (DU26), Shenmen (HT7), Neiguan (PC6), Fengchi (GB20), Wangu (GB12), Tianzhu (BL10), Shenshu (BL23), Taichong (LR3), Fenglong (ST40)			12 w	②, ④
Liu Runli 2017 [19]	32	32	Electroacupuncture + Usual therapy	Usual therapy	Baihui (DU20), Shenting (DU24)	56.9 ± 10.3	56.4 ± 10.1	2 w	①
Liu Xiaoxiao 2015 [20]	14	14	Acupuncture + Nimodipine	Nimodipine	Baihui (DU20), Sishencong (EX-HN1), Sibai (ST2), Fengchi (GB20), Wangu (GB12), Tianzhu (BL10), Shenmen (HT7), Neiguan (PC6), Shuigou (DU26), Sanyinjiao (SP6), Taichong (LR3), Fenglong (ST40), Qihai (RN6), Xuehai (SP10), Geshu (BL17), Zusanli (ST36)	63.86 ± 9.07	61.79 ± 4.96	12 w	②
Luo Jianchang 2019 [21]	94	94	Electroacupuncture	Nimodipine	Sihua, Fengchi (GB20), Fengfu (DU16), Dazhui (DU14)	71 ± 7	70 ± 7	8 w	②,③, ⑤
Peng Juan 2022 [22]	35	35	Acupuncture + Ganglioside	Ganglioside	Zusanli (ST36), Guanyuan (RN4), Baihui (DU20), Sishencong (EX-HN1)	61.31 ± 7.23	61.60 ± 7.55	2 w	②③ ④
Sun Jia 2018 [23]	50	50	Acupuncture + Cognitive training	Cognitive training	Acupoint selection based on syndromic differentiation	64.38 ± 8.39	65.11 ± 8.23	12 w	①②③ ④
Tang Liang 2022 [24]	20	20	Acupuncture + Donepezil	Donepezil	Jin's three-needle technique	70.20 ± 9.83	64.60 ± 8.55	12 w	① ③
Wang Fang 2014 [25]	30	30	Acupuncture + Nimodipine	Nimodipine	Baihui (DU20), Shenting (DU24), Sishencong (EX-HN1), Hegu (LI4), Taichong (LR3), Sanyinjiao (SP6), Fenglong (ST40), Zhongwan (RN12), Zusanli (ST36)			12 w	②
Wang Lingfei 2018 [26]	64	64	Acupuncture + Nimodipine	Nimodipine	Shuigou (DU26), Neiguan (PC6), Sanyinjiao (SP6)	71.42 ± 8.67	69.33 ± 7.56	10 w	② ③
Wang Qin 2019 [27]	59	59	Acupuncture + Atorvastatin	Atorvastatin	Baihui (DU20), Fengchi (GB20), Shenting (DU24), Fengfu (DU16), Dazhui (DU14), Jingjiaji (EX-B2), Quchi (LI11), Neiguan (PC6), Fengshi (GB31), Zusanli (ST36), Yanglingquan (GB34), Sanyinjiao (SP6), Xuehai (SP10)	68.88 ± 3.64	67.71 ± 3.02	4 w	①, ③
Wang Yan 2021 [28]	30	30	Electroacupuncture + Cognitive training	Cognitive training	Baihui (DU20), Shenting (DU24), Neiguan (PC6), Shenmen (HT7)	53.6 ± 9.9	52.5 ± 13.4	4 w	②, ④
Xie Yaqing 2021 [29]	31	30	Acupuncture + Donepezil	Donepezil	Baihui (DU20), Sishencong (EX-HN1), Shenting (DU24), Ganshu (BL18), Shenshu (BL23), Taixi (KI3), Xuanzhong (GB39), Sanyinjiao (SP6), Zusanli (ST36)	65.90 ± 8.35	66.03 ± 8.86	4 w	①,②,③, ④
Yang Hongling 2015 [30]	36	36	Acupuncture + Nimodipine	Nimodipine	Baihui (DU20), Sishencong (EX-HN1), Sibai (ST2), Fengchi (GB20), Wangu (GB12), Tianzhu (BL10), Shenmen (HT7), Neiguan (PC6), Shuigou (DU26), Sanyinjiao (SP6), Taichong (LR3), Fenglong (ST40)	65.89 ± 3.28	65.97 ± 3.31	12 w	①, ②
Yang Jinhua 2022 [31]	35	35	Acupuncture + Usual therapy	Usual therapy	Baihui (GV20), Fengfu (DU16), Dazhui (DU14), Dazhu (BL11), Shangjuxu (ST37), Xiajuxu (ST39), Zusanli (ST36)	64.6 ± 13.0	65.5 ± 14.0	8 w	①,②, ③
Yang Weining 2021 [32]	30	30	Acupuncture + Nimodipine	Nimodipine	Baihui (DU20), Shenting (DU24), Benshen (BG13), Taixi (KI3), Sishencong (EX-HN1), Sanyinjiao (SP6), Zusanli (ST36)	74.48 ± 9.37	74.19 ± 9.51	6 w	①,②, ③
Yuan Hongwei 2022 [33]	39	40	Acupuncture + Cognitive training	Cognitive training	Baihui (DU20), Shenting (DU24), Shendao (DU11), Fengfu (DU16), Xinshu (BL15)	54.3 ± 7.7	68.7 ± 5.0	4 w	②,③, ④
Yujin Choi 2021 [34]	20	19	Electroacupuncture	Sham acupuncture	Baihui (DU20), Sishencong (EX-HN1), Shenting (DU24), Neiguan (PC6), Zusanli (ST36), Taixi (KI3), Shenmen (HT7)	64.1	64.1	12 w	②
Zeng Youhua 2015 [35]	50	50	Electroacupuncture	Cognitive training	Baihui (DU20), Sishencong (EX-HN1), Shenting (DU24), Yintang (DU29), Hegu (LI4), Taichong (LR3)	66 ± 12	68 ± 10	8 w	②, ⑤
Zhang Han 2021 [36]	23	23	Electroacupuncture + Cognitive training	Cognitive training	Baihui (DU20), Sishencong (EX-HN1), Shenting (DU24), Benshen (BG13), Neiguan (PC6), Shenmen (HT7)	61 ± 9	60 ± 8	4 w	②,③, ④
Zhang Jinfeng 2015 [37]	30	30	Acupuncture + Nimodipine	Nimodipine	Baihui (DU20), Taiyang (EX-HN5), Xinshu (BL15), Ganshu (BL18), Mingmen (DU4), Neiguan (PC6), Shenmen (HT7), Hegu (LI4), Zhongwan (RN12), Qihai (RN6), Zusanli (ST36), Xuehai (SP10), Sanyinjiao (SP6), Taichong (LR3), Taixi (KI3)	64.03 ± 6.23	63.60 ± 6.41	8 w	②, ③
Zhang Jiyun 2018 [38]	30	30	Acupuncture + Nimodipine	Nimodipine	Shenshu (BL23), Guanyuan (RN4), Fengfu (DU16), Shenmen (HT7), Taixi (KI3), Shousanli (LI10), Zusanli (ST36), Shuigou (DU26), Shenting (DU24), Chengling (GB18), Fenglong (ST40)	58.17 ± 6.54	59.03 ± 6.06	4 w	②, ⑤
Zhang Xiaojian 2017 [39]	42	42	Acupuncture + Atorvastatin	Atorvastatin	Baihui (DU20), Fengfu (DU16), Yamen (DU15), Shenting (DU24), Shuigou (DU26), Dazhui (DU14), Jingjiaji (EX-B2)	63.28 ± 10.68	63.07 ± 10.59	4 w	①, ②
Zhao Ling 2012 [40]	94	93	Electroacupuncture	Nimodipine	Baihui (DU20), Sishencong (EX-HN1), Shenting (DU24), Fengchi (GB20)	69 ± 7	67 ± 6	8 w	①, ③
Zhou Junying 2019 [41]	60	60	Acupuncture + Cognitive training	Cognitive training	Baihui (DU20), Shenting (DU24)	61.44 ± 8.77	62.04 ± 8.69	4 w	①,②, ③

① total effective rate, ② MoCA score, ③ MMSE score, ④ ADL score, ⑤ Barthel index score.

### 2.3 Quality evaluation of the included studies

**Fig. 2:**
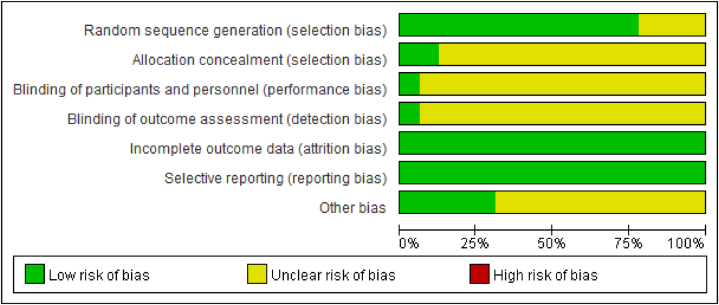
Assessment of the risk of bias in the included studies.

A total of 32 randomized controlled trials were included in this study. The results of the study quality evaluation were as follows. ① Random sequence generation: Twenty-five studies used the random number table method to generate random sequences; these studies were rated as having a low risk of bias for this item. The other 7 studies merely mentioned the word “random”, from which we could not judge the risk level; these studies were rated as uncertain. ② Allocation concealment: Three studies used sealed opaque envelopes to hide the random allocations; these studies were evaluated as having a low risk of bias for this item. The remaining 29 studies did not mention the method of allocation concealment; their risk level was considered uncertain. (3) Blinding of participants and personnel: Only 2 studies mentioned the use of double blinding; these studies were evaluated as low risk for this item. The remaining 30 studies did not mention the implementation of subject or researcher blinding; these studies were deemed to have an uncertain risk level. (4) Blinding of outcome assessment: All studies failed to mention this item, and their risk level was evaluated as uncertain; (5) Data integrity (incomplete outcome data and selective reporting): All of the included studies reported their data in full and specified the reasons for any withdrawals or losses to follow-up; thus, all the studies were evaluated as low risk. (6) Other bias: Reported adverse events were used as the main form of outcome evaluation in this review. A total of 10 studies clearly reported the occurrence of adverse events and were therefore evaluated as low risk; the remaining 22 studies were evaluated as uncertain risk. The risk-of-bias assessment of the included studies is illustrated in Fig. 2.

### 2.4 Meta-analysis of acupuncture for VMCI

#### 2.4.1 Total effective rate

**Fig. 3:**
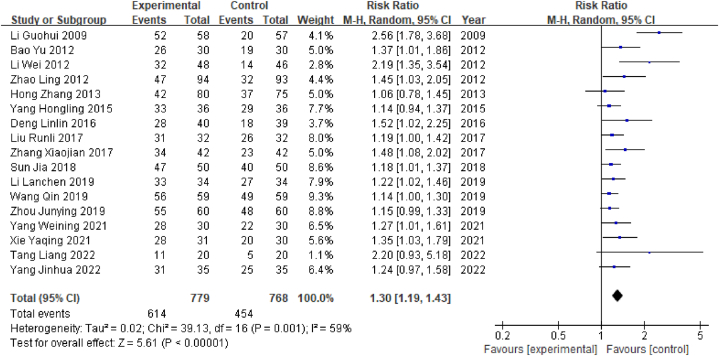
Forest plot for overall effective rates.

Total effective (response) rates were reported in 17 of the 32 studies included in this study. A total of 1547 patients were enrolled, comprising 779 in the experimental group and 768 in the control group. In terms of efficacy, the total number of responders in the experimental group was 614, and the total number of responders in the control group was 454. Since this outcome index was a dichotomous variable, the RR value was selected. Heterogeneity test results showed that P < 0.05 and I^2^ = 59% (I^2^ < 50%), indicating that the heterogeneity between studies was high; therefore, the random-effects model was selected. The results of the meta-analysis showed that RR = 1.30, 95% CI [1.19, 1.43] (P < 0.05), indicating a significant difference in the effective rate between the observation group and the control group; specifically, acupuncture had a significantly higher overall effective rate than Western medicine alone in the treatment of MCI after stroke (Fig. 3).

#### 2.4.2 MoCA scores

**Fig. 4:**
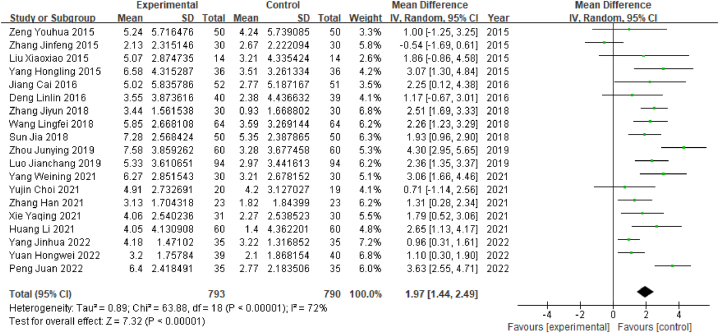
Forest plot for MoCA scores.

Among the 32 studies included in this review, a total of 20 studies reported MoCA scores, among which 1 study reported only the total score, and another study did not report the standard deviation; therefore, no combined analysis could be conducted. A total of 1583 patients were enrolled in the remaining 19 studies, comprising 793 in the experimental group and 790 in the control group. As the outcome index was a continuous variable, the WMD value was used. The heterogeneity test results showed that P < 0.00001 and I^2^ = 72% (I^2^ > 50%), indicating that the heterogeneity between studies was high; therefore, the random effect model was selected. The results of the meta-analysis showed that WMD = 1.97, 95% CI [1.44, 2.49] (P < 0.05), indicating that there was a significant difference in the effective rate between the acupuncture group and the control group; specifically, acupuncture was associated with significantly higher MoCA scores than Western medicine among poststroke MCI patients (Fig. 4).

#### 2.4.3 Subgroup analysis of MoCA scores by acupuncture type

**Fig. 5:**
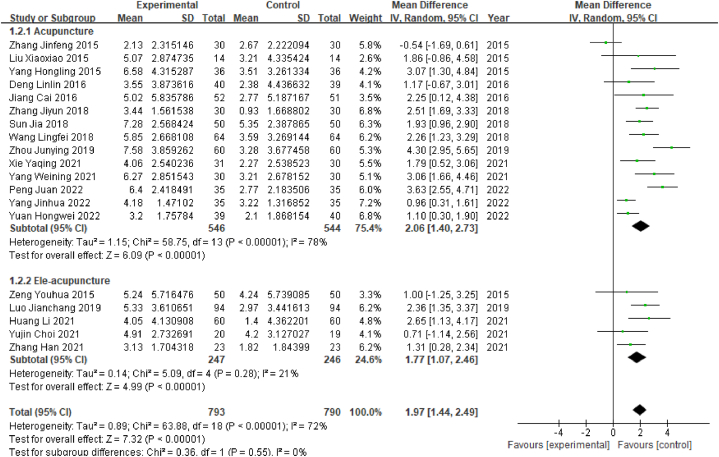
Forest plot for subgroup analysis of MoCA scores by acupuncture type.

The heterogeneity analysis showed that the degree of heterogeneity was high. Further sensitivity analysis and heterogeneity analysis were conducted to explore the source of heterogeneity. Sensitivity analysis showed that I^2^ = 63% after the study by Zhang Jinfeng (2015) was excluded, indicating that this study had a certain impact on heterogeneity. In the subgroup analysis of acupuncture types, as shown in Fig. 5, heterogeneity tests revealed especially high heterogeneity in the ordinary acupuncture subgroup (14 studies): P < 0.001, I^2^ = 78%. The meta-analysis within that subgroup showed that WMD = 2.06, 95% CI [1.40, 2.73] (P < 0.05), indicating that the ordinary acupuncture treatment subgroup had significantly better MoCA scores than the control group. For the electroacupuncture subgroup, the meta-analysis showed that WMD = 1.77, 95% CI [1.07, 2.46] (P < 0.05), indicating that the electroacupuncture subgroup had significantly better MoCA scores than the control group (Fig. 5).

#### 2.4.4 Subgroup analysis of MoCA scores by duration of treatment

**Fig. 6:**
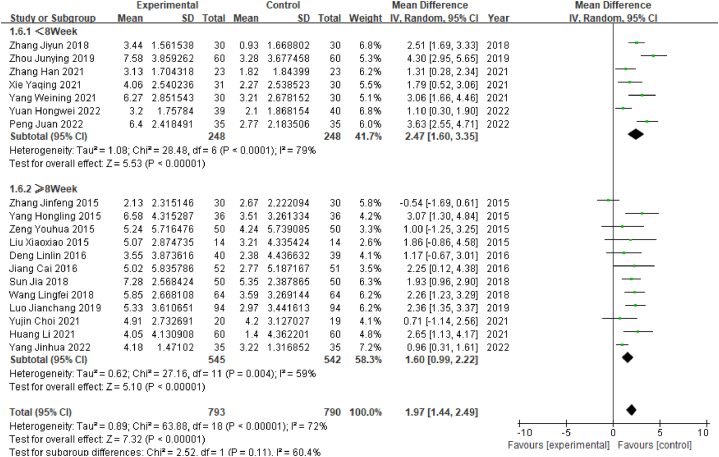
Forest plot for subgroup analysis of MoCA scores by duration of treatment.

Among the 19 studies that collected MoCA scores, the shortest course of treatment was 2 weeks, and the longest course was 12 weeks. On the basis of treatment duration, the studies were divided into two subgroups: the long-course subgroup (≥8 weeks), which contained 12 studies, and the short-course subgroup (<8 weeks), which contained 7 studies. Heterogeneity tests of the short-course subgroup revealed high heterogeneity (P < 0.05, I^2^ = 79%). The meta-analysis for that subgroup showed that WMD = 2.47, 95% CI [1.60, 3.53] (P < 0.05), indicating that a short course of acupuncture was associated with significantly better MoCA scores than a control treatment in poststroke MCI patients. Similarly, the heterogeneity tests of the long-course subgroup revealed high heterogeneity (P < 0.05, I^2^ = 59%), and the meta-analysis showed that WMD = 1.60, 95% CI [0.99, 2.22] (P < 0.05), indicating that the subgroup treated with a long course of acupuncture for MCI had significantly better MoCA scores than the control group (Fig. 6).

#### 2.4.5 MMSE scores

**Fig. 7:**
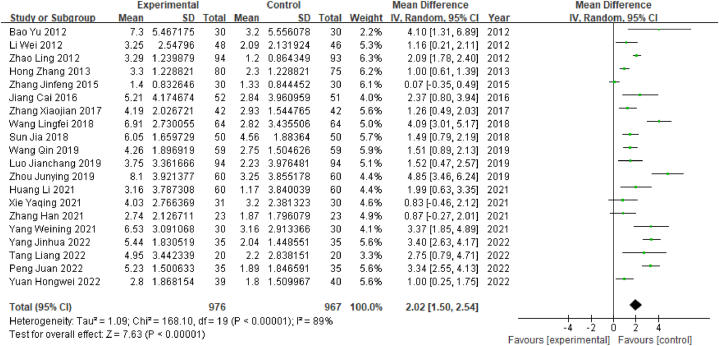
Forest plot of MMSE scores.

A total of 21 studies reported MMSE scores, among which 1 study reported only the separate subscores and not the total score; thus, a combined analysis was not possible. A total of 1943 patients were included in the remaining 20 studies, comprising 976 in the experimental group and 967 in the control group. The outcome index was a continuous variable; therefore, the WMD value was used. Heterogeneity test results showed that P < 0.001 and I^2^ = 89% (I^2^ > 50%), indicating a high degree of heterogeneity; therefore, the random-effects model was selected. The results of the meta-analysis showed that WMD = 2.02, 95% CI [1.50, 2.54] (P < 0.05), indicating that acupuncture treatment was associated with significantly higher MMSE scores than Western medicine in poststroke MCI patients (Fig. 7).

##### 2.4.5.1 Subgroup analysis of MMSE scores by acupuncture type

**Fig. 8:**
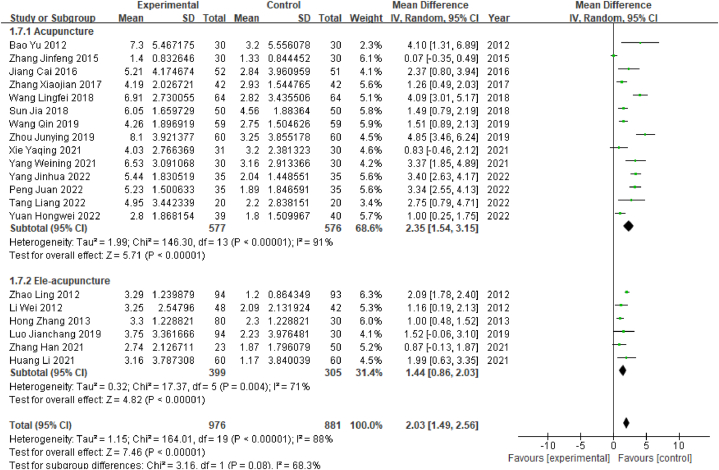
Forest plot for subgroup analysis of MMSE scores by acupuncture type.

According to the type of acupuncture performed, 20 studies were divided into an ordinary acupuncture subgroup and an electroacupuncture subgroup. Heterogeneity tests for the ordinary acupuncture subgroup (14 studies) revealed a high degree of heterogeneity (P < 0.001, I^2^ = 92%; I^2^ > 50%). The meta-analysis for that subgroup showed that WMD = 2.35, 95% CI [1.54, 3.15] (P < 0.05), indicating that the acupuncture treatment subgroup had significantly better MMSE scores than the control subgroup. In the electroacupuncture subgroup (6 studies), significant heterogeneity was found (P < 0.05, I^2^ = 71%, I^2^ > 50%). The meta-analysis showed that WMD = 1.44, 95% CI [0.86, 2.03] (P < 0.05), indicating that the EA treatment subgroup had significantly better MMSE scores than the control subgroup (Fig. 8).

##### 2.4.5.2 Subgroup analysis of MMSE scores by duration of treatment

**Fig. 9:**
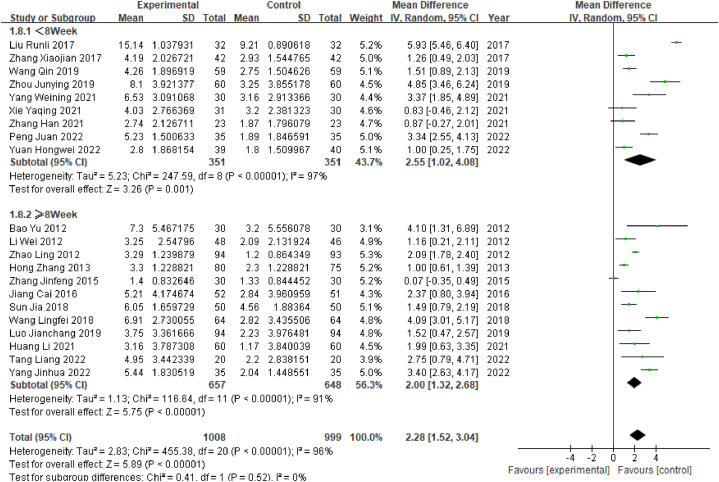
Forest plot for subgroup analysis of MMSE scores by duration of treatment.

On the basis of treatment duration, the studies were divided into two subgroups: the long-course subgroup (≥8 weeks), which contained 11 studies, and the short-course subgroup (<8 weeks), which contained 9 studies. Heterogeneity tests for the short-course group revealed a high degree of heterogeneity (P < 0.05, I^2^ = 97%). The meta-analysis showed that WMD = 2.55, 95% CI [1.02, 4.08] (P < 0.05), indicating that MCI patients treated with a short course of acupuncture had significantly better MMSE scores than the control group. For the long-course group, tests also showed a high degree of heterogeneity: P < 0.05, I^2^ = 91%. The meta-analysis showed that WMD = 2.00, 95% CI [1.32, 2.68] (P < 0.05), indicating that MCI patients treated with a long course of acupuncture achieved significantly better MMSE scores than the control group (Fig. 9).

#### 2.4.6 Barthel index

**Fig. 10:**
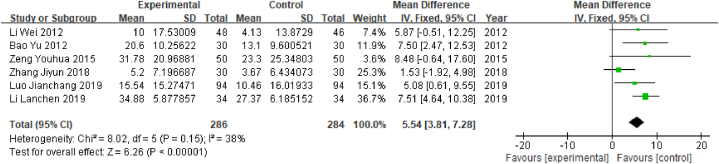
Forest plot of Barthel index values.

In this review, the Barthel index was reported in 6 studies, which contained a total of 570 patients: 286 in the experimental group and 284 in the control group. The outcome index was a continuous variable; therefore, the WMD value was used. Heterogeneity tests showed that P > 0.05 and I^2^ = 38% (I^2^ < 50%), indicating that the degree of heterogeneity was low; therefore, the fixed-effects model was selected. The results of the meta-analysis showed that WMD = 5.54, 95% CI [3.81, 7.28] (P < 0.05), indicating that acupuncture was associated with significantly better Barthel index values than Western medicine among poststroke MCI patients (Fig. 10).

#### 2.4.7 ADL scores

**Fig. 11:**
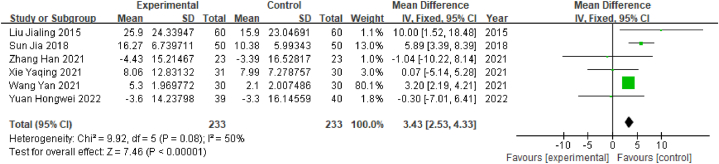
Forest plot of ADL scores.

A total of 6 studies reported ADL scores as a measure of quality of life; together, these studies included 466 patients, comprising 233 in the experimental group and 233 in the control group. The outcome index was a continuous variable; therefore, the WMD value was used. Heterogeneity tests showed that P > 0.05 and I^2^ = 50%, indicating that there was no significant heterogeneity among studies; therefore, the fixed-effects model was selected. The results of the meta-analysis showed that WMD = 3.43, 95% CI [2.53, 4.33] (P < 0.05), indicating that poststroke MCI patients treated with acupuncture achieved significantly better ADL scores than those who were treated with Western medicine (Fig. 11).

#### 2.4.8 Publication bias

**Fig. 12:**
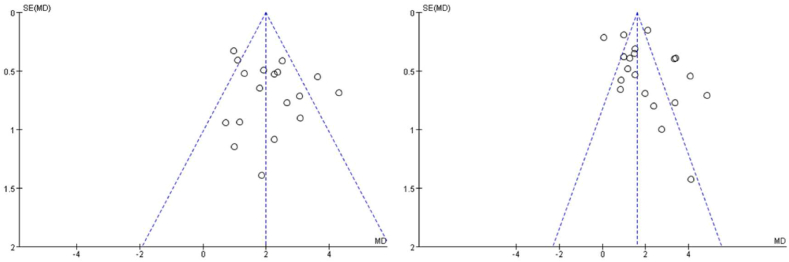
Funnel plots to test for publication bias (MoCA scores on the left, MMSE scores on the right).

In this study, publication bias was evaluated for the main indicators of MoCA and MMSE scores, and RevMan 5.3 software was used to draw the corresponding funnel plots. In the funnel plot for each of the two variables, the points corresponding to individual studies were symmetrically distributed on both sides of the central axis (Fig. 12), indicating that there was no obvious publication bias.

In addition to the qualitative method of funnel plots, the quantitative analysis methods of Egger's test and Begg's test were used to further evaluate the publication bias of studies within this meta-analysis. The analysis results showed that when MoCA scores were the variable of interest, both methods showed P > 0.05, meaning that no unacceptable publication bias was found. When MMSE scores were the variable of interest, Egger's and Begg's tests once again showed P > 0.05, indicating that there was no significant publication bias (Table 2).

**Table 2:** Egger's and Begg's test results.

Test Method	MoCA	MMSE
Egger's test	0.674	0.622
Begg's test	0.675	0.673

**Fig. 13:**
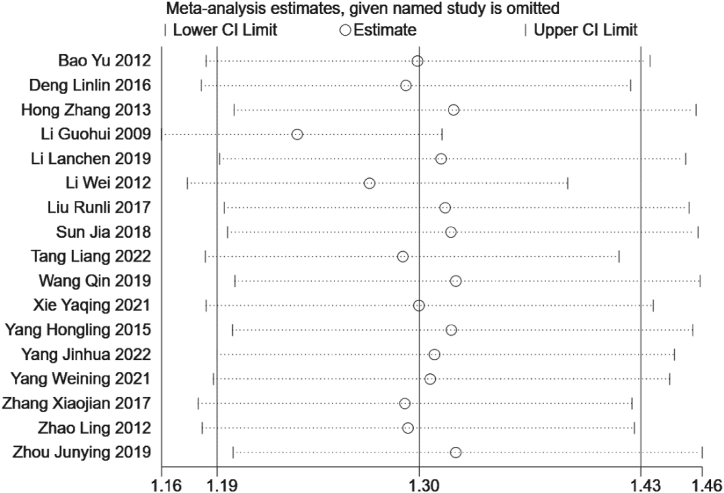
Sensitivity analysis interval chart.

Sensitivity analysis was performed using the leave-one-out approach. The results showed that the RR value of the total effective rate of the 17 included studies ranged from 1.16 to 1.46 after excluding any one study, which was the same as the results of the overall meta-analysis. Therefore, the results of the meta-analysis of this study were relatively stable (Fig. 13).

### 2.5 Meta-analysis of electroacupuncture for VMCI

#### 2.5.1 Total effective rate

**Fig. 14:**
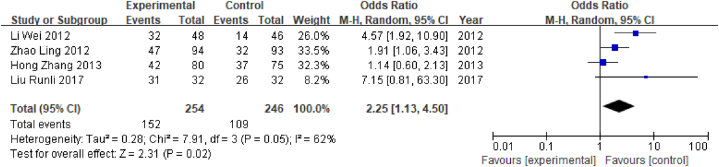
Forest plot for total effective rate.

In this study, a total of 10 studies used electroacupuncture as an intervention, of which 4 studies reported the total effective rate. A total of 400 patients were enrolled, comprising 254 in the experimental group and 246 in the control group. Treatment was effective in 152 cases in the experimental group and 109 cases in the control group. This outcome index was a dichotomous variable; therefore, the RR value was selected. Heterogeneity test results showed that P = 0.02 and I^2^ = 62% (I^2^ > 50%), indicating that there was pronounced heterogeneity; therefore, the random-effects model was selected. Meta-analysis showed that RR = 2.25, 95% CI [1.13, 4.50] (P < 0.05), indicating that the total effective rate of the acupuncture group was significantly better than that of the control group (Fig. 14).

#### 2.5.2 MoCA scores

**Fig. 15:**
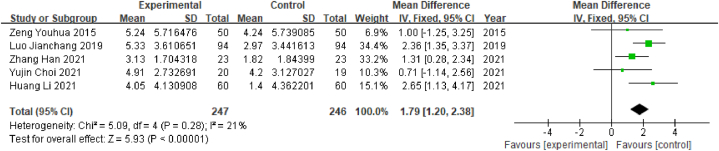
Forest plot for MoCA scores.

MoCA scores were reported in 5 of the 8 electroacupuncture studies included in this review. A total of 493 patients were enrolled, comprising 247 in the experimental group and 246 in the control group. The outcome index was a continuous variable; therefore, the WMD value was used. Heterogeneity test results showed that P = 0.28 and I^2^ = 21% (I^2^ < 50%), indicating that the heterogeneity between studies was low; therefore, the fixed-effects model was selected. The results of the meta-analysis showed that WMD = 1.79, 95% CI [1.20, 2.38] (P < 0.05), indicating that there was a significant difference between the observation group and the control group, indicating that electroacupuncture was associated with significantly better MoCA scores than Western medicine in poststroke MCI patients (Fig. 15).

#### 2.5.3 MMSE scores

**Fig. 16:**
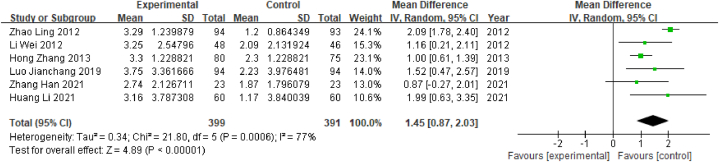
Forest plot for MMSE scores.

A total of 6 studies evaluated MMSE scores; these studies included a total of 790 patients, comprising 399 in the experimental group and 391 in the control group. Heterogeneity tests showed that P < 0.001 and I^2^ = 77% (I^2^ > 50%), indicating the presence of heterogeneity; therefore, the random-effects model was used. The meta-analysis showed that WMD = 1.45, 95% CI [0.87, 2.03] (P < 0.05), indicating that there was a significant difference between the observation group and the control group, indicating that the electroacupuncture treatment group had significantly better MCI scores than the control group (Fig. 16).

#### 2.5.4 Barthel index

**Fig. 17:**
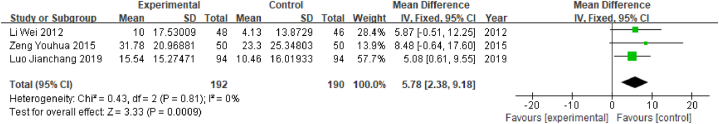
Forest map of Barthel index scores.

A total of 3 studies reported Barthel index values; together, those studies included 382 patients, comprising 192 in the experimental group and 190 in the control group. The outcome index was a continuous variable; therefore, the WMD value was used. Heterogeneity tests showed that P > 0.05 and I^2^ = 0% (I^2^ < 50%), indicating that there was no heterogeneity among the studies; therefore, the fixed-effects model was selected. The results of the meta-analysis showed that WMD = 5.78, 95% CI [2.38, 9.18] (P < 0.05), indicating that poststroke MCI patients treated with electroacupuncture treatment achieved significantly better Barthel index scores than those treated with Western medicine (Fig. 17).

#### 2.5.5 ADL scores

**Fig. 18:**
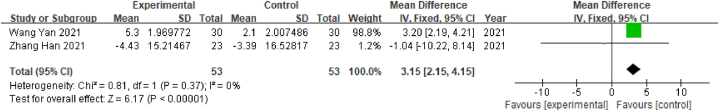
Forest map of ADL scores.

In this review, 2 studies reported ADL scores as a measure of quality of life; those studies enrolled a total of 106 patients, comprising 53 patients in the experimental group and 53 patients in the control group. The outcome index was a continuous variable; therefore, the WMD value was used. Heterogeneity tests showed that P > 0.05 and I^2^ = 0% (meeting the 0% threshold), indicating that there was no heterogeneity among the studies; therefore, the fixed-effects model was selected. The meta-analysis showed that WMD = 3.15, 95% CI [2.15, 4.15] (P < 0.05), indicating that poststroke MCI patients treated with electroacupuncture achieved better ADL scores than those treated with Western medicine (Fig. 18).

#### 2.5.6 Publication bias

In this study, publication bias was evaluated for the main observation indexes of MoCA and MMSE scores. Since the number of studies using each index was less than 10, funnel plots were not appropriate; only quantitative analysis methods (Egger's and Begg's tests) were used to evaluate publication in this meta-analysis. When the MoCA score was used as the detection index, both detection methods showed that P > 0.05, meaning that no unacceptable publication bias was found. Similarly, when MMSE scores were used as the observation index, Egger's and Begg's tests both showed that P > 0.05, indicating that there was no significant publication bias (Table 3).

**Table 3:** Egger's and Begg's test results.

Test Method	MoCA	MMSE
Egger's test	0.607	0.906
Begg's test	0.462	0.707

## 3 Discussion

This review included 32 randomized controlled trials. In a total of 2808 subjects, the overall effective (response) rates and the posttreatment changes in MoCA scores, MMSE scores, Barthel index values and ADL scores were significantly more favourable in people treated with acupuncture than in those treated with other therapies alone, such as simple drug therapy, thus indicating that acupuncture improved cognitive function in subjects with MCI. Subgroup analysis showed that the MoCA and MMSE scores of the electroacupuncture and long-course acupuncture subgroups in particular were better than those of the control group. Funnel plots did not reveal any unacceptable publication bias. Meanwhile, comparisons of the posttreatment changes in MoCA, MMSE, Barthel index and ADL scores showed that electroacupuncture was superior to other therapies alone, such as simple drug therapy, in the treatment of VMCI.

At present, the clinical drugs for vascular cognitive impairment are mainly brain cell activators and cerebral vasodilators, which are often used in conjunction with neuroimmunomodulators, hydrogenated ergot, antioxidants, nerve growth factor and other drugs [42]. Clinical research results show that these drugs improve cognitive function in patients with different degrees of symptom severity and ADL capacity, but their effect is not ideal. However, it is notable that on November 29, Eisai announced at the CATD conference in San Francisco that a phase 3 study of Lecanemab, an anti-beta amyloid (Aβ) antibody, found that treatment with Lecanemab for 18 months, compared with the placebo group, led to a 27% reduction in the Clinical Dementia Rating Scale Sum of Boxes (CDR-SB) scores as well as improvements in other secondary endpoints, thus leading to new hopes for drug treatment of cognitive disorders [43].

Traditional Chinese acupuncture, as a typical nondrug therapy, has been shown to improve pain (cancer pain [44], chronic headache [45], chronic low back pain [46], etc.), chronic prostatitis/chronic pelvic pain syndrome [47], prophylaxis of episodic migraine without aura [48] and the treatment of cognitive disorders have been clinically proven effective. Acupuncture has been clinically demonstrated to be effective in improving cognitive impairment. MoCA scores show high sensitivity and specificity in the diagnosis of VMCI [49]. In addition, the MMSE score is not only the most sensitive marker for VMCI diagnosis but also the most sensitive marker for dementia diagnosis [50]. Therefore, the MMSE and MoCA scores are the main indexes used to evaluate patients' cognitive function, with higher scores indicating better cognitive ability. The results of our study also show that acupuncture therapy is significantly more effective than nonacupuncture therapy in improving MMSE and MoCA scores; comprehensive treatment that combines acupuncture and drugs may benefit patients most in clinical practice. Additionally, in terms of the Barthel index and the ADL scale, acupuncture therapy significantly outperformed nonacupuncture therapy in improving VMCI patients’ ability to function in daily life, which can effectively improve their quality of life.

Our results also showed that electroacupuncture was more effective than conventional acupuncture in improving MMSE and MoCA scores. In experimental studies using rats with septic brain injury and rats with cognitive impairment induced by limb ischaemia‒reperfusion, electroacupuncture treatment was reported to reduce inflammation, oxidative stress, and cell apoptosis. Electroacupuncture treatment has been shown to reduce cytokine levels in the inflamed hippocampus, prolong the necrotic phase of pyramidal cells, and reduce acetylcholinesterase (AChE) activation in a rat model of vascular dementia [51,52]. Recent studies have also found that electroacupuncture can drive the vagus-adrenal axis to inhibit inflammation in the body [53]. Previous studies by our team have also found that electroacupuncture can inhibit the inflammatory cascade by inhibiting the NLRP3/caspase-1 pathway to trigger hippocampal neuron pyroptosis and improve cognitive function [54]. Additionally, other studies have shown that electroacupuncture upregulates autophagy, downregulates Notch signalling, and increases adenosine monophosphate–activated protein kinase (AMPK), which is closely associated with cognitive function as the primary energy sensor in Alzheimer's disease rats [55,56]. Similarly, the effects of electroacupuncture on inflammation, stress, enzymes, signal transduction pathways, and direct activation of antioxidants and specific enzymes in the hippocampus can be inferred as factors underlying the mechanisms by which this therapy affects cognitive impairment and simultaneously prevents cognitive decline [57].

The findings of the present review should be interpreted with caution due to a number of limitations. First, while we did not exclude articles published in other languages during our search, all randomized controlled trials that met the criteria were conducted in China, and a large proportion of them were published in Chinese; therefore, the findings may be biased. Second, the specific acupoints differed between studies. The main acupoints were generally similar across most studies, but different acupoints were used in individual studies. Third, the acupuncture treatments included in this study were not consistent, although subgroup analyses showed that a variety of acupuncture protocols yielded statistically significant improvements compared with nonacupuncture treatments. However, it is not clear whether different acupuncture methods have the same effect. Fourth, some of the included studies had small sample sizes; therefore, clinical trials with larger sample sizes are needed to validate their results. Finally, the diagnostic criteria in previous studies have not been consistent. Several diagnostic criteria were cited in this study, which allowed a degree of randomness in the inclusion of the literature.

This study analysed effective rates, MoCA scores and MMSE scores by systematic evaluation and showed that acupuncture therapy improved the cognitive function of patients with VMCI, which provides an evidentiary basis for the clinical promotion and application of acupuncture for this condition. However, the diagnostic criteria and evaluation indicators for vascular cognitive dysfunction need to be further standardized and unified.

The results of this systematic review and meta-analysis should be interpreted with caution because of the small sample sizes in some of the analyses and the limited geographic range of the studies. Future clinical studies should adopt a high-quality double-blind randomized controlled trial design, large multicentre samples, long-term efficacy evaluation, and rigorous scientific methodology. In addition, animal experimental studies should be carried out to further explore the mechanism through which acupuncture mitigates VMCI.

## Author contribution statement

All authors listed have significantly contributed to the development and the writing of this article.

## Data availability statement

Data will be made available on request.

## Declaration of competing interest

The authors declare that they have no known competing financial interests or personal relationships that could have appeared to influence the work reported in this paper.
